# Catalpol ameliorates psoriasis-like phenotypes via SIRT1 mediated suppression of NF-κB and MAPKs signaling pathways

**DOI:** 10.1080/21655979.2020.1863015

**Published:** 2020-12-31

**Authors:** Aimin Liu, Buxin Zhang, Wei Zhao, Yuanhui Tu, Qingxing Wang, Jing Li

**Affiliations:** a Department of Dermatology, Henan Province Hospital of Traditional Chinese Medicine, The Second Affiliated Hospital of Henan University of Chinese Medicine, Zhengzhou, People’s Republic of China

**Keywords:** Catalpol, psoriasis, oxidative stress, inflammatory response, silent information regulator 1, nuclear factor kappa B, mitogen-activated protein kinases

## Abstract

Psoriasis is a chronic inflammatory skin disease that affects approximately 2% of worldwide population, and causing long-term troubles to the patients. Therefore, it is urgent to develop safe and effective therapeutic drugs. Catalpol is a natural iridoid glucoside, that has several remarkable pharmacological effects, however, whether catalpol can alleviated psoriasis has not been explored. The goal of the present work is to study the role of catalpol in psoriasis in vivo and in vitro. Imiquimod-induced psoriasis-like mice were applied with different concentrations of catalpol for 8 consecutive days. The severity degree of psoriasis was estimated and the skin pathological changes were detected by H&E staining. Also, TNF-α-stimulated keratinocytes were treated with different concentrations of catalpol, then the oxidative stress and inflammation factors, as well as the expression of SIRT1 and activation of NF-kB and MAPK pathways were measured. The results showed that catalpol reduced the erythema, scaling, ear thickness, and changed pathological phenotypes in the lesioned skin region in mice. Treatment with catalpol significantly suppressed the oxidative stress and inflammatory reactions *in vivo* and *in vitro*, as reflected by the decreased secretion or expression of oxidative stress indicators and proinflammatory factors. Furthermore, the SIRT1 was up-regulated and the NF-κB and MAPKs signaling pathways were suppressed by the treatment of catalpol *in vivo* and *in vitro*. In summary, our data suggested that catalpol may have a therapeutic property of psoriasis by ameliorating oxidative stress and inflammation partly through SIRT1 mediated suppression of NF-κB and MAPKs pathways.

**Abbreviation:** CAT: catalase; ELISA: enzyme-linked immunosorbent assay; GSH: glutathione; HRP: horseradish peroxidase; IMQ: imiquimod; JNK: c-Jun NH 2-terminal kinases; MAPKs: mitogen-activated protein kinases; MDA: malondialdehyde; NC: negative control group; NF-kB: nuclear factor kappa B; PASI: psoriasis area and severity index; PVDF: polyvinylidene difluoride membranes; qRT-PCR: quantitative real time polymerase chain reaction; ROS: reactive oxygen species; SDS-PAGE: sodium dodecyl sulfate-polyacrylamide gel; SIRT1: silent information regulator 1; SOD: Cu/Zn superoxide dismutase

## GRAPHICAL ABSTSRACT

**Figure:**
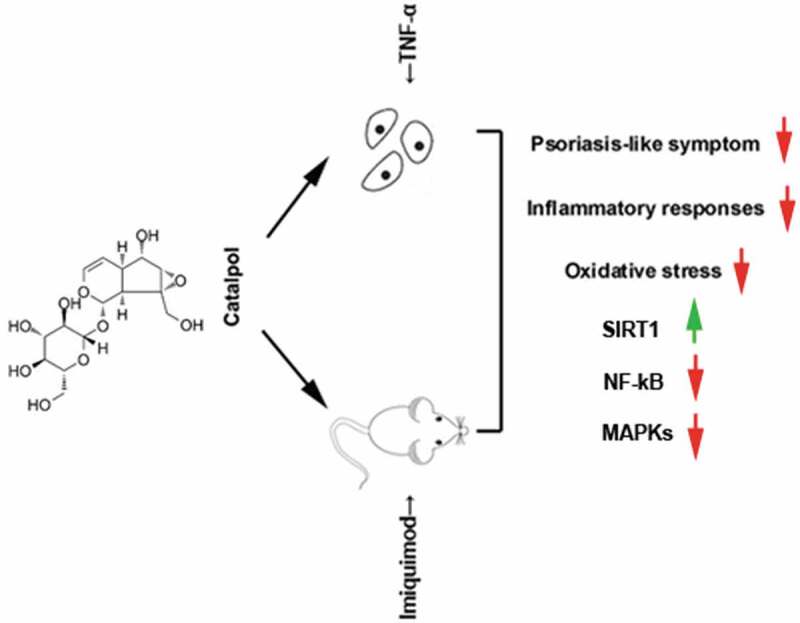

## Introduction

Psoriasis is a chronic inflammatory disease with autoimmune pathogenic traits and a strong genetic predisposition. The prevalence is about 2% worldwide [1]. It has a life-long process of the chronic relapsing treatment course, which imposes a substantial economic burden on patients. Psoriasis is characterized by abnormal keratinization, epidermal hyper-proliferation, and inflammation [2,3]. The pathogenesis of psoriasis is not fully understood. Although several biologics, conventional systemic therapies, or drugs have been clinically proven efficient, their uses are associated with a higher cost, systemic toxicity, or increased risk of lymphoma and skin cancer [4–6]. Therefore, it is crucial to explore safe and effective novel therapeutic drugs to treat psoriasis.

Catalpol is an iridoid glucoside isolated from the roots of *Rehmannia glutinosa*. Studies have reported that catalpol has several remarkable pharmacological effects, including anti-diabetic [7,8], anti-tumor [9,10], anti-hypoglycemic [11], and anti-asthma [12] properties. Especially, its anti-oxidant and anti-inflammatory effects were found in a variety of experimental studies *in vitro* and *in vivo* [13–16]. Zhang et al. suggested that catalpol alleviated inflammation reactions in LPS-stimulated bovine endometrial epithelial cells via the suppression of the TLR4/NF-kB signaling pathway [17]. Catalpol suppressed homocysteine-induced oxidation and inflammation in human aorta endothelial cells [18]. Catalpol also attenuated liver fibrosis by the repress of the inflammation response in CCl_4_-exposed rats [19]. Administration of catalpol ameliorated high-fat diet-induced adipose tissue inflammation by inhibition of the JNK and NF-kB pathways [16]. Since the inflammatory reaction is the major feature of psoriasis, we speculate that catalpol may be able to alleviate the psoriasis symptoms through its anti-inflammatory activity.

The present study aims to investigate the effect of catalpol on psoriasis *in vivo* and *in vitro*. We found that catalpol could alleviate psoriasis-like responses, and these effects are related to the antioxidant stress and anti-inflammatory properties of catalpol. Here, the beneficial effects of catalpol in ameliorating psoriasis have been firstly demonstrated.

## Materials and methods

### Experimental animals

Male BALB/c mice (Liaoning Changsheng, Benxi, China), at 8–11 weeks of age, were anesthetized with 3% pentobarbital sodium (50 mg/kg) and shaved of hair on their back and left ear. The animals were randomly divided into 5 groups: the negative control group (NC), the imiquimod treatment group (IMQ), and imiquimod plus different concentrations of catalpol (Cas No. 2415–24-9, Aladdin, Shanghai, China). The control mice were left untreated. The other mice were administrated with 62.5 mg IMQ cream (MED SHINE, Chengdu, China) daily on the shaved skin. Two days before IMQ cream was applied, mice in the IMQ+CAT-L group, IMQ+CAT-M group, and IMQ+CAT-H group were intraperitoneally injected with 2.5 mg/kg, 5 mg/kg, or 10 mg/kg catalpol for 8 consecutive days. The mice were anesthetized with 200 mg/kg pentobarbital sodium on the seventh day, and the skin samples were collected for subsequent experiments. According to the Psoriasis Area and Severity Index (PASI) objective scoring system, the total scores (erythema, scaling, and thickness) were recorded to estimate the severity degree of psoriasis. All animal experiments were approved by the Ethics Committee of Henan Province Hospital of Traditional Chinese Medicine and following the Guide for the Care and Use of Laboratory Animals.

### H&E staining

The skin tissues from each group were fixed in 4% paraformaldehyde and embedded in paraffin and cut into 5 μm-thickness following the standard procedures[21]. Then the sections were stained with H&E staining solution as previously reported [20].

### Quantitative real-time PCR (qRT-PCR)

The RNA from skin tissues was isolated by using the RNA simple Total RNA Kit (TIANGEN, Beijing, China). Then the RNA was converted into cDNA using M-MLV reverse transcriptase (TIANGEN, Beijing, China). The qRT-PCR reaction system including: 1 μl cDNA, 0.3 μl SYBR Green (Solarbio, Beijing, China), 10 μl 2× Taq PCR MasterMix (TIANGEN, Beijing, China), 0.5 μl sense primer, 0.5 μl antisense primer, then make up the total volume to 20 μl with ddH_2_O. The PCR reaction was set as follows: 94 °C 5 min; 94 °C 10 s, 60 °C 20 s, 72 °C 30 s, 40 cycles; 72 °C 2.5 min, 40 °C 2.5 min, melting from 60 °C to 94 °C, incubate at 25 °C. The reaction was conducted in the Exicycler TM 96 (Bioneer, Daejeon, Korea). The sequences of the PCR primers were described as follows: 5ʹ-CAGGCGGTGCCTATGTCTCA-3ʹ (sense) and 5ʹ-GCTCCTCCACTTGGTGGTTT-3ʹ (antisense) for mus TNF-α; 5ʹ-ATGGCAATTCTGATTGTATG-3ʹ (sense) and 5ʹ-GACTCTGGCTTTGTCTTTCT-3ʹ (antisense) for mus IL-6; 5ʹ-CTCAACTGTGAAATGCCACC-3ʹ (sense) and 5ʹ-GAGTGATACTGCCTGCCTGA-3ʹ (antisense) for mus IL-1β; 5‘-AAACACTGAGGCCAAGGAC-3ʹ (sense) and 5ʹ-CGTGGAACGGTTGAGGTAG-3ʹ (antisense) for mus IL-17A; 5ʹ-GACAGGTTCCAGCCCTACAT-3ʹ (sense) and 5ʹ-CAGCCTTCTGACATTCTTCT-3ʹ (antisense) for mus IL-22; 5ʹ-TGTTCCTACCCCCAATGTGTCCGTC-3ʹ (sense) and 5ʹ-CTGGTCCTCAGTGTAGCCCAAGATG-3ʹ (antisense) for mus GAPDH; 5ʹ- GACCTGACCTGCCGTCTAG-3ʹ (sense) and 5ʹ-AGGAGTGGGTGTCGCTGT-3ʹ (antisense) for homo GAPDH. GAPDH levels were used for normalization of the mRNA levels.

### Cell culture

HaCaT cell line was purchased from Procell (Wuhan, China). The cells were cultured in MEM (Gibco, Grand Island, NY, USA) medium supplemented with 15% fetal bovine serum at 37 °C, 5% CO_2_. 0.2% trypsin solution was employed for cell digestion and passage. The cells were passaged every 2–3 days. In the catalpol treatment group, cells were incubated with different concentrations of catalpol for 24 h.

### Enzyme-linked immunosorbent assay (ELISA)

The IL-1β, TNF-α, IL-6, IL-22, IL-17A, or ICAM-1 levels were measured by commercial kits purchased from MULTI SCIENCES (Hangzhou, China). In brief, the psoriatic tissues were homogenized and centrifuged, the supernatant of HaCaT cells was collected, then the protein concentrations were respectively determined by the BCA protein determination kit (Solarbio, Beijing, China). The proteins were diluted to 1 mg/ml, next, the standard curve was established as per the user’s manuals, then the OD value was detected with a microplate spectrophotometer (ELx-800, Biotek Instruments, VT, USA).

### CCK-8 assay

HaCaT cells were seeded into 96-well culture plates (5 × 10^3^ cells/well) and incubated with different concentrations of catalpol for 24 h. After that, 10 μL of CCK-8 working solution was added, and the cells were cultured at 37 °C for another 2 h. The absorbance at 450 nm was assessed by a microplate reader (ELX-800, BIOTEK, Biotek Winooski, Vermont, USA).

### Detection of intracellular ROS

For the measurement of the ROS level, HaCaT cells were cultured in 6-well plates. The cells were incubated with different concentrations of catalpol for 12 h and then stimulated with 10 ng/mL of TNF-α for 12 h. At the end of the administration, the medium of each well was replaced with 1 mL of DCFH-DA diluent (diluted with a serum-free medium at 1:1000) and incubated for 30 min. At last, the fluorescence intensity was detected by a flow cytometer (NovoCyte, ACEABIO, San Diego, California, USA).

### Oxidative stress detection

To estimate the oxidative stress level of the psoriatic tissues of mice or HaCaT cells, we applied a series of commercial kits (Nanjing Jiancheng Bioengineering Institute, Nanjing, China) to exam the activity or concentration of SOD, MDA, GSH, and CAT following the instruction manuals. In short, after the tissues or cells were lysed, the protein concentration of each sample was calculated according to the standard curve and diluted with normal saline to a uniform concentration of 1 g/L. Next, each indicator was tested according to the instructions.

### Western blot assay

Equal amounts of proteins were electrophoresed on the SDS-PAGE (10%, 12%) and then electrically transferred onto the polyvinyl difluoride (PVDF) membranes. The primary antibodies used are as follows: p-JNK (1:1000 dilution, CST, Danvers, Massachusetts, USA) JNK (1:1000 dilution, CST, Danvers, Massachusetts, USA), p-ERK (1:500 dilution, CST, Danvers, Massachusetts, USA) ERK (1:1000 dilution, CST, Danvers, Massachusetts, USA), p-IkB (1:1000 dilution, CST, Danvers, Massachusetts, USA), IkB (1:1000 dilution, CST, Danvers, Massachusetts, USA), p-p65 (1:1000 dilution, CST, Danvers, Massachusetts, USA), p65 (1:1000 dilution, CST, Danvers, Massachusetts, USA), p-p38 (1:400 dilution, Bioss, Woburn, Massachusetts, USA), p38.(1:500 dilution, Bioss, Woburn, Massachusetts, USA), and GAPDH (1:10,000 dilution, Proteintech, Wuhan, China), SIRT1 (1: 1000 dilution, ABclonal, Wuhan, China). The membranes were incubated with different primary antibodies at 4 °C overnight. The secondary antibody HRP-conjugated goat anti-mouse (Solarbio, Beijing, China) or HRP-conjugated goat anti-rabbit (Solarbio, Beijing, China) was incubated with membranes at 37 °C for 1 h. The protein bands were visualized by ECL plus (Solarbio, Beijing, China). GAPDH was used as an internal control for gray analysis.

### Statistical analysis

All these experiments were repeated at least in triplicate. The data are presented as mean ± SD. The differences among groups were analyzed by using One-Way ANOVA. p-values<0.05 were considered statistically significant.

## Results

1. Catalpol alleviates IMQ-induced psoriasis-like symptoms of mice.

**Figure 1.**
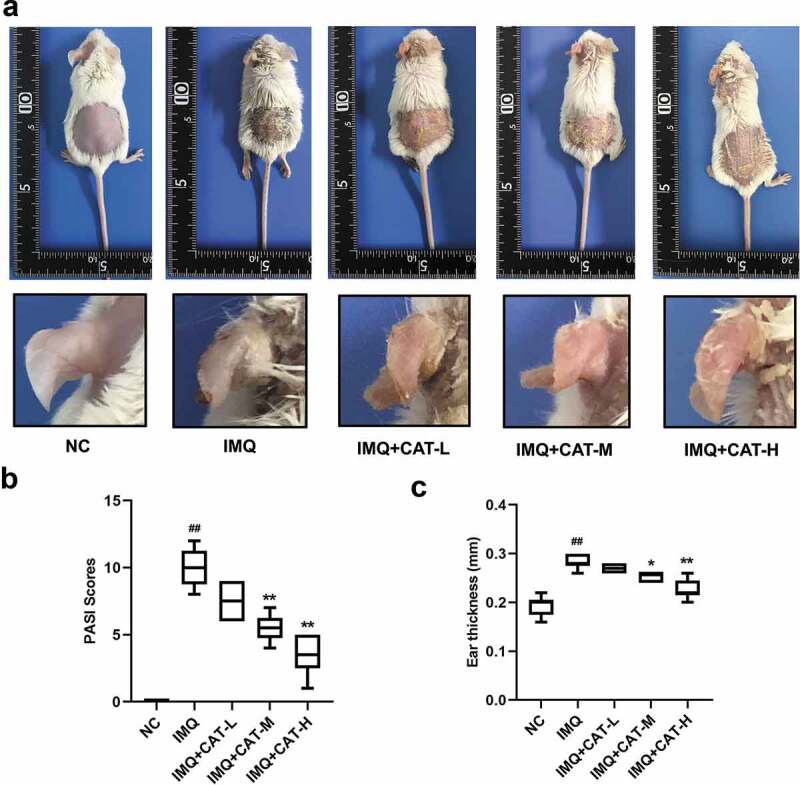
The role of catalpol on IMQ-induced psoriatic-like symptoms *in vivo.*

**Figure 2.**
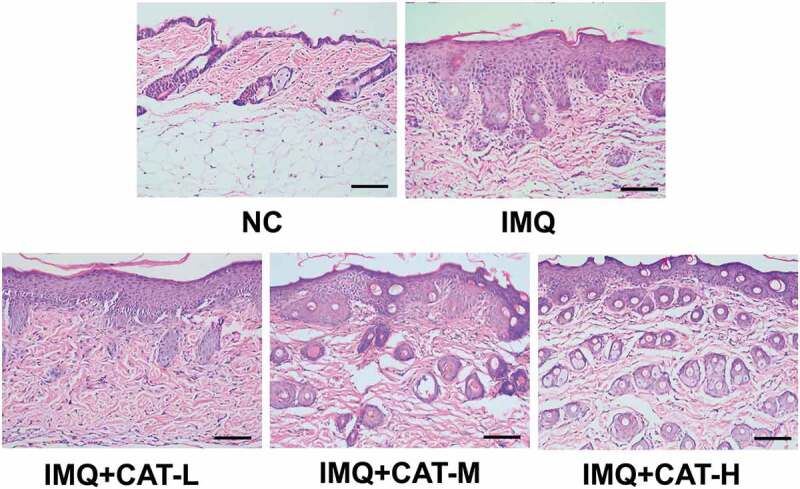
The role of catalpol on the IMQ-induced pathological injury was analyzed by H&E staining. Scale bar = 200 μm. The typical images of 6 repetitions from each group

We employed IMQ-induced psoriasis-like mice model to evaluate the effect of catalpol on psoriasis. IMQ cream was administrated on the shaved skin of mice, which led to psoriasis-like symptoms such as scaling, erythema, and thickening (Figure 1(a)). However, catalpol treatment (5 mg/kg and 10 mg/kg) ameliorated the phenotypic changes and reduced the PASI scores induced by IMQ application as observed in Figure 1(a,b). We also measured the thickness of the left ears of mice in each group. The 5 mg/kg or 10 mg/kg concentrations of catalpol dramatically reduced the ear thickness of psoriatic-like mice (Figure 1(c)). The results of histological analyses depicted severe acanthosis in the epidermis and increased epidermal hyperplasia in mice treated with IMQ, while these were obviously mitigated by the treatment of catalpol in a dose-dependent manner (Figure 2). These results suggested that catalpol treatment could alleviate IMQ-induced psoriasis-like symptoms in mice.

(a). Catalpol (CAT-L: 2.5 mg/kg, CAT-M: 5 mg/kg, CAT-H: 10 mg/kg) administration alleviated the psoriatic phenotypes of mice. (b). Scaling, redness, and thickness based PASI scores were recorded on day 7. (c). Ear thickness of each mouse was measured on day 7. Data were presented as mean± SD. ^##^p < 0.01 *vs*. NC group. *p < 0.05 and **p < 0.01 *vs*. the IMQ group.

2. Catalpol inhibits oxidative stress and inflammatory response in psoriasis-like skin tissues of mice.

**Figure 3.**
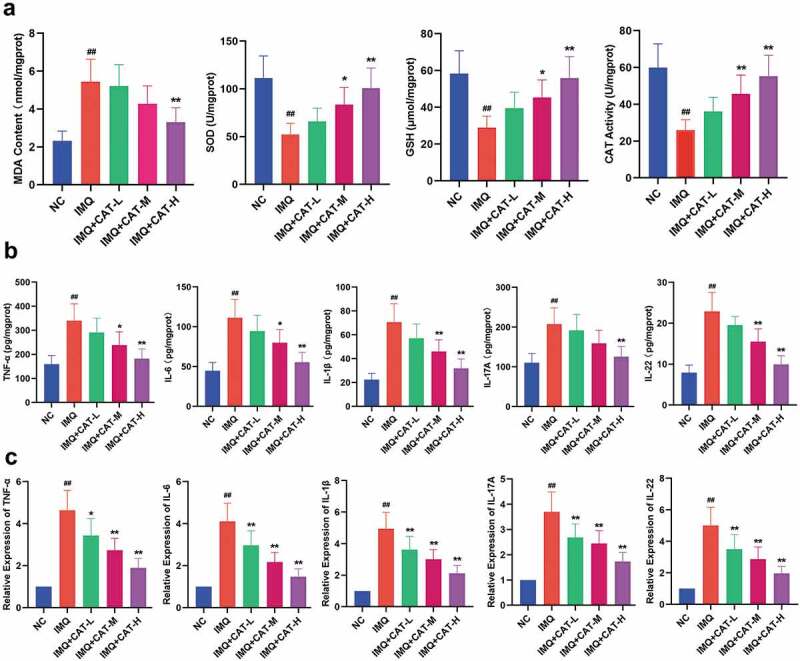
The effect of catalpol on oxidative stress markers and inflammatory cytokines in psoriatic-like skin tissues. (a). Catalpol decreased the MDA level, elevated the GSH level and the SOD, CAT activities. (b). Catalpol treatment ameliorated inflammatory cytokine productions caused by the IMQ application. The cytokine levels were measured by ELISA. (c). Catalpol treatment decreased the mRNA expression levels of IL-6, TNF-α, IL-1β, IL-22, and IL-17A, in psoriatic-like skin tissues. The expression levels were quantified by qRT-PCR. Data were presented as mean± SD. ^##^p < 0.01 *vs*. the NC group. *p < 0.05 and **p < 0.01 *vs*. the IMQ group

In this study, the levels of MDA, GSH, SOD, and CAT in the lesioned skin tissues were determined by commercial kits. As shown in Figure 3(a), the IMQ group showed a higher level of MDA and lower levels of GSH, SOD, and CAT compared to mice in the control group. Nevertheless, catalpol injection effectively reversed the enhanced oxidative stress in psoriatic model mice. The administration of IMQ led to the proliferation of keratinocytes through elevating various inflammatory cytokines [21]. Therefore, we measured the levels of several proinflammatory cytokines in the IMQ-induced psoriatic skin lesion by ELISA and qRT-PCR. The ELISA results showed that the levels of IL-6, TNF-α, IL-1β, IL-22, and IL-17A were significantly increased in the IMQ group compared with untreated mice. Administration of catalpol strongly suppressed these IMQ-induced elevations in a dose-dependent manner (Figure 3(b)). Similarly, the injection of catalpol dramatically suppressed the mRNA expression levels of IL-6, TNF-α, IL-1β, IL-22, and IL-17A induced by IMQ in mice (Figure 3(c)). Based on these results, catalpol dose-dependently inhibits oxidative stress and inflammatory response in psoriasis-like skin tissues.

3. Catalpol inhibits the NF-κB and MAPKs signaling pathways and stimulated SIRT1 expression in psoriasis-like mice.

**Figure 4.**
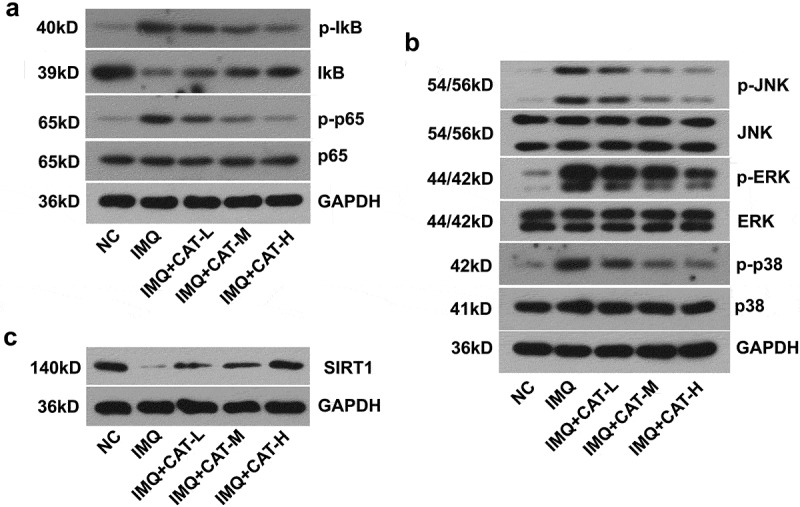
The effect of catalpol on the expression of SIRT1, NF-κB, and MAPKs signaling pathways in psoriatic-like mice. (a). The level of p-IkB, IkB, p-p65, and p65 was measured by western blot. (b). The level of p-JNK, JNK, p-ERK, ERK, p-p38, and p38 was examined by western blot. (c). The expression of SIRT1 was measured by western blot. Data were demonstrated as mean± SD. ^##^p < 0.01 *vs*. NC group. *p < 0.05 and **p < 0.01 *vs*. the IMQ group

Oxidative stress is implicated in the alteration of the inflammatory cascade, such as NF-κB and MAPKs pathways. To delineate the molecular mechanism by which catalpol suppresses the production of inflammatory cytokines induced by IMQ, a western blot was utilized. It was observed that treatment with 2.5 mg/kg, 5 mg/kg, or 10 mg/kg catalpol repressed the expression of p-IkB and p-p65 and enhanced the expression of IkB in IMQ stimulated skin tissues (Figure 4(a)). On the other side, the IMQ application strongly promoted the activation of three MAPKs, ERK, JNK, and p38. In contrast, catalpol treatment led to an obvious decrease in the expression of p-JNK, p-ERK, and p-p38 in mice compared with the IMQ group (Figure 4(b)). Additionally, IMQ induced loss of SIRT1 in the skins was restored by the treatment of catalpol (Figure 4(c)). Our data suggested that catalpol inhibited the NF-κB and MAPKs signaling pathways and up-regulated the expression of SIRT1 in the skin of psoriasis-like mice.

4. Catalpol inhibits TNF-α-induced oxidative stress and inflammation reactions in human keratinocytes.

**Figure 5.**
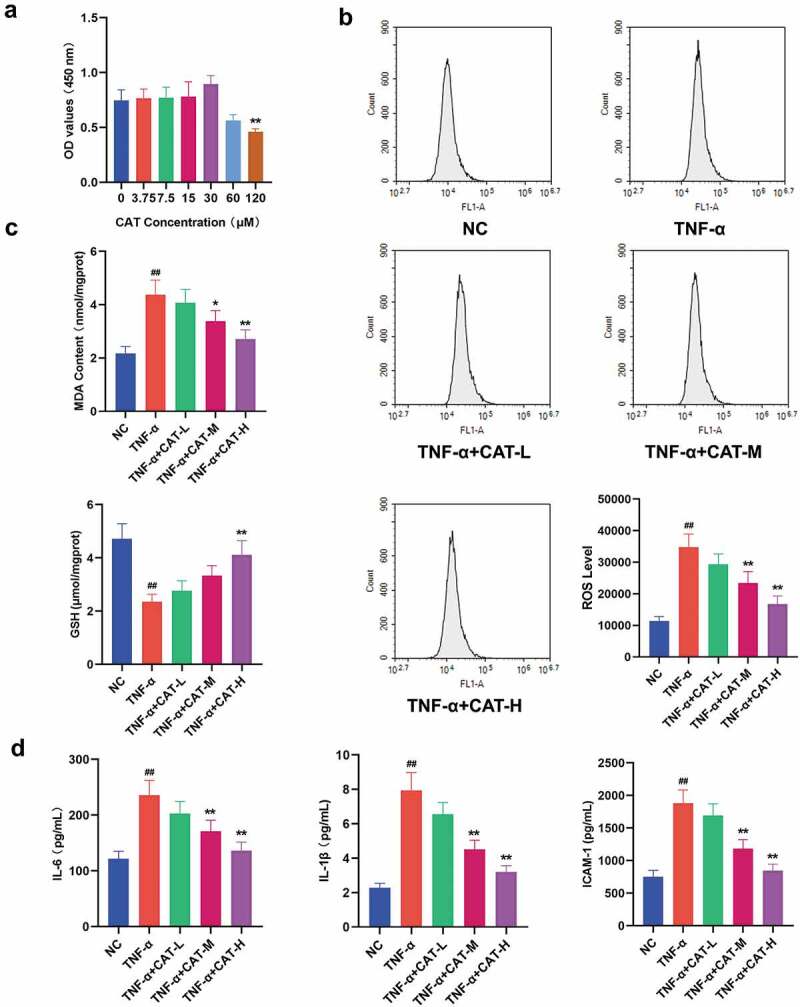
The role of catalpol on oxidative stress and inflammation responses in human keratinocytes. (a). CCK-8 assay was performed to detect cell viability after treatment with 0 μM, 3.75 μM, 7.5 μM, 15 μM, 30 μM, 60 μM, and 120 μM catalpol for 24 h. (b). HaCaT cells were pretreated with 7.5 μM, 15 μM, or 30 μM catalpol for 12 h, then the cells were treated with 10 ng/mL TNF-α for 12 h, then the ROS generation was measured by flow cytometry. (c). Effect of catalpol on the levels of MDA and GSH in the supernatants of HaCaT cells. (d). Effect of catalpol on the concentrations of IL-6, IL-1β, and ICAM-1 in the supernatants. Data were demonstrated as mean± SD. ^##^p < 0.01 *vs*. the NC group. *p < 0.05 and **p < 0.01 *vs*. the TNF-α group

The CCK-8 results showed that catalpol at 0–30 μM has no cytotoxicity for keratinocytes, and 60–120 μM catapol reduced the viability of keratinocytes (Figure 5(a)). Based on the maximum concentration that catapol does not affect cell viability, catalpol of 7.5 μM, 15 μM, and 30 μM were used to treat TNF-α-induced HaCaT cells. Excessive ROS led to oxidative damage to a variety of cell types [23]. As shown in Figure 5(b), TNF-α stimulation resulted in an obvious increase of DCF-fluorescence in HaCaT cells, which indicates the up-regulation of the ROS level. However, treatment with catalpol significantly reduced the generation of ROS in TNF-α simulated cells. As demonstrated in Figure 5(c), the increased MDA level and reduced GSH level induced by TNF-α were strikingly attenuated by catalpol. The ELISA data showed that the concentrations of IL-6, IL-1β, and ICAM-1 in the supernatants of HaCaT cells stimulated with TNF-α were significantly increased. However, treatment with 15 μM and 30 μM catalpol markedly reduced the concentration of IL-6, IL-1β, and ICAM-1 (Figure 5(d)). These results indicated that catalpol suppresses the oxidative stress and inflammatory response in keratinocytes.

5. Catalpol inhibits NF-κB and MAPKs signaling pathways and up-regulated SIRT1 expression of TNF-α-induced human keratinocytes.

**Figure 6.**
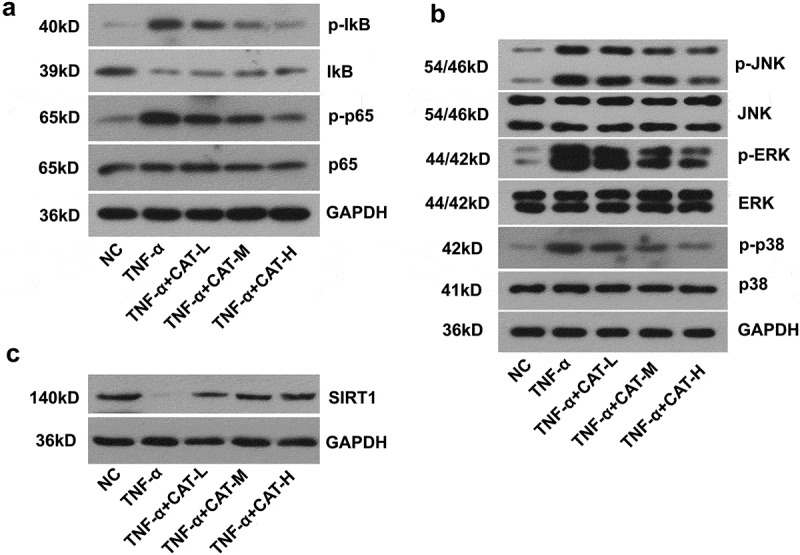
The regulation of catalpol on the expression of SIRT1, NF-κB, and MAPKs signaling pathways in TNF-α-induced keratinocytes. (a). The expression of p-IkB, IkB, p-p65, and p65 proteins was measured by western blot. (b). The level of p-JNK, JNK, p-ERK, ERK, p-p38, and p38 proteins was measured by western blot. (c). The level of SIRT1 was detected by western blot. Data were presented as mean± SD. ^##^p < 0.01 *vs*. the NC group. *p < 0.05 and **p < 0.01 *vs*. the TNF-α group

To evaluate the effect of catalpol on TNF-α-stimulated HaCaT cells, the expression of proteins implicated in NF-κB and MAPKs signaling pathways was determined. The data showed that catalpol blocked TNF-α-induced activation of the NF-kB pathway in a dose-dependent manner (Figure 6(a)). As shown in Figure 6(b), catalpol intervention dose-dependently led to a significant decrease in the relative expression of p-JNK, p-ERK, and p-p38 that activated by TNF-α (Figure 6(b)). Further, TNF-α induced reduction of SIRT1 expression was also counteracted by the treatment of catalpol (Figure 6(a)). These results suggested that catalpol inhibits the activities of NF-κB and MAPKs signaling pathways and rescued the SIRT1 expression in TNF-α-induced keratinocytes.

## Discussion

Psoriasis is a chronic skin disease involving multiple cell types and is associated with activated immune responses. Epidermal keratinocytes are the primary cell type implicated in skin injury. They can recruit immune cells to the injured tissues by the secretion of various factors, such as cytokines, chemokines, or adhesion molecules, resulting in an escalation of the inflammatory response [22]. It is well accepted that herbal medicines may play key roles and provide new therapeutic approaches in the treatment of inflammatory skin diseases [23,24]. However, safe and effective therapeutic drugs still need to be explored.

The topical application of IMQ to mouse skin could provoke inflammatory lesions [25]. IMQ may exert its function through the adenosine receptor on keratinocytes, which results in the secretion of the pro-inflammatory cytokines [26]. We sought to explore the inflammation severity by the PASI score. Our results showed that catalpol treatment alleviated the erythema, scaling, and ear thickness in the lesioned skin region of mice in a dose-dependent manner. The results of the histopathological analysis demonstrated that catalpol treatment remarkably reduced the epidermal layer at the injury skin of psoriatic-like mice.

The complex interaction between pathogenic T cells and dendritic cell-driven immune systems, which exacerbates the inflammation of psoriasis, resulting in uncontrolled proliferation of keratinocytes. The pro-inflammatory cytokines, such as IL-6, TNF-α, IL-1β, IL-22, and IL-17A, have been reported to be up-regulated in psoriatic lesioned tissue and serum, and these cytokines likely drive the psoriasis pathogenesis [27]. In our study, a strong increase of the IL-6, TNF-α, IL-1β, IL-22, and IL-17A, was observed in mice with IMQ application. The pretreatment of catalpol exerted a prominent inhibitory effect on the production of these cytokines in psoriasis-like skin tissues *in vivo*. ICAM-1, which could be strongly induced by proinflammatory cytokines in various cell types, plays a pivotal role in immune cell recruitment to the skin [28]. ICAM-1 provides adhesion sites between neutrophils and lymphocytes and activates vascular endothelial cells to release inflammatory mediators, and it may contribute to psoriasis [29]. In this work, we found that catalpol suppressed TNF-α-induced IL-1β, IL-6, and ICAM-1 secretion in keratinocytes *in vitro*, which suggests that catalpol could suppress inflammation response in skin disease.

A dynamic balance of oxidants and antioxidants exists in healthy skin, and any imbalance can lead to skin diseases, including psoriasis [30,31]. A large number of studies indicated that using various compounds with antioxidant effects to increase the content of SOD, CAT, and GSH and eliminate ROS could effectively alleviate the symptoms of psoriasis [32]. Our study found that the activities of SOD, GSH, and CAT were significantly higher in psoriatic mice treated with catalpol, and the content of MDA, which reflected the level of lipid peroxidation, was notably decreased. ROS is released and removed by the ROS elimination system under normal physiological conditions, thereby maintaining cellular redox balance. The aberrant ROS was reported to exert a harmful effect on psoriasis, which provides a continuous self-sustaining circulatory system for psoriasis in aggravating inflammation through highly interconnected clusters. Catalpol treatment also attenuated the ROS activity of TNF-α-stimulated keratinocytes, reduced the MDA level, and elevated the GSH level in the supernatant of inflammatory keratinocytes culture. The above results suggest that catalpol partly alleviates psoriasis by improving oxidative stress.

In the last decades, a variety of drugs or compounds have been reported to down-regulate the associated signaling pathways to reduce the release of inflammatory factors in keratinocytes [33,34]. NF-κB and MAPKs (JNK, ERK, and p38) signaling contribute to the inflammatory cascade reactions that cause epidermal hyperplasia and the psoriatic-like phenotype [35–37]. NF-κB is a crucial contributor that participated in the regulation of inflammatory pathways and a key mediator in the progress of psoriasis [38]. High levels of TNF-α related to NF-κB activation was observed in psoriatic patients [39]. NF-κB signaling is activated when the keratinocytes are stimulated with TNF-α, which caused the increased expression and secretion of a variety of pro-inflammatory cytokines. Applying with the NF-kB antagonist BAY-11-7082 obviously blunted epidermal thickness, acanthosis, and inflammatory in imiquimod-induced psoriasis-like dermatitis in mice [35]. Additionally, several studies suggested that the repression of the NF-kB pathway can effectively alleviate the psoriasis symptoms *in vitro* and *in vivo* [40–42]. In this work, we demonstrated that catalpol could effectively inhibit IkB degradation and p65 phosphorylation in psoriatic lesioned skin and TNF-α-stimulated keratinocytes.

The MAPKs, JNK, ERK, or p38 may take different functions in regulating the expression of pro-inflammatory factors and the pathogenesis of psoriasis. For instance, MMP-9 induced by TNF-α relies on the activation of JNK in keratinocytes [43]. The suppression of ERK and p38 blocked the release of ICAM-1 stimulated by TNF-α at mRNA and protein levels [44]. Xiong et al [45]. found that glycyrrhizin ameliorated imiquimod-induced psoriasis-like skin lesions in mice and TNF-α-induced ICAM-1 expression in HaCaT cells by the suppression of MAPKs. Other studies also suggested that the suppression of p38 [46,47], JNK [48,49] or ERK [50,51] signaling pathway all contribute to the protection of psoriasis associated symptoms. In the present work, we found that pretreatment of catalpol decreased IMQ-induced or TNF-α-stimulated excitation of ERK, JNK, and p38 in a dose-dependent manner in psoriatic lesioned skin or human keratinocytes, respectively. Therefore, the beneficial effect of catalpol on psoriasis was at least partly dependent on its suppressive activities on NF-κB and MAPKs signaling pathways.

SIRT1 is a member of the deacetylases family sirtuins (SIRTs), which functions as molecular sensors of nutritional status in organisms and participate in regulating cellular oxidative stress or inflammatory burdens [52]. Studies have suggested that the expression of SIRT1 was reduced under chronic inflammatory or oxidative stress environments [53]. SIRT1 could counteract the activation of NF-kB pathways or MAPK proinflammatory pathways by its deacetylases activities [54], thus maintaining or activation of SIRT1 expression can effectively resolve various inflammatory diseases [55,56], especially in the psoriasis-like symptoms [20,57,58]. A recent study found that catalpol efficiently binds to the active site of SIRT1 and functions as a SIRT1 activator [59], this effect was later confirmed by other teams [60]. In the present study, the treatment of catalpol obviously up-regulated SIRT1 level and inactivated the NF-kB and MAPK pathways in psoriasis conditions *in vitro* and *in vivo*, indicating that catalpol may improve the psoriasis-like symptoms via SIRT1 mediated anti-inflammatory effects.

It is known that skin diseases such as psoriasis are mainly treated by external application in the clinic. As a preliminary study, the catalpol was injected intraperitoneally in the present work. In this way, the drug is intestinal absorbed and circulated to the lesion site, it may not be as effective as the subcutaneous injection or surface smearing for psoriasis. There is a limitation that the delivery method of catalpol more suitable for clinical treatment is not explored. It is necessary to investigate the effect of catalpol cream on psoriasis in the future study.

## Conclusion

**Figure 7.**
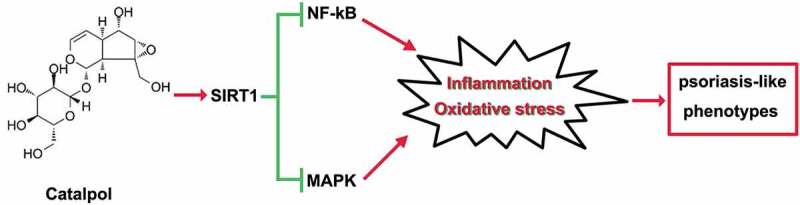
The mechanism on how catalpol mitigated psoriatic-like symptoms. Catalpol up-regulated the expression of SIRT1, the activated SIRT1 restrained the inflammatory activities and oxidative stress by the inactivation of NF-kB and MAPK pathways in vivo and in vitro

In conclusion, catalpol treatment ameliorated psoriatic-like symptoms *in vivo* and *in vitro*. These effects are at least partly related to the up-regulation of SIRT1, which suppressed the inflammatory response and oxidative stress via blocking the inflammatory-associated signaling cascade, NF-κB, and MAPKs signaling pathways (Figure 7). The present study suggests the protective effect of catalpol on psoriasis, and our results provide a potential alternative treatment strategy for the clinical treatment of psoriasis.

## Supplementary Material

Supplemental Material

## Research Highlights

- Catalpol alleviated Imiquimod-induced psoriasis-like symptom in vivo.
- Catalpol suppressed TNF-α-induced oxidative stress in keratinocytes.
- Catalpol suppressed TNF-α-induced inflammatory responses in keratinocytes.
- Catalpol blocked the NF-κB and MAPKs pathways via the up-regulation of SIRT1.
