# Supplementary figures and images for: Catalpol ameliorates psoriasis-like phenotypes via SIRT1 mediated suppression of NF-κB and MAPKs signaling pathways
Source: Bioengineered. 2020 Dec 31;12(1):183–95. doi: 10.1080/21655979.2020.1863015 (PMC8806253; doi:10.1080/21655979.2020.1863015)

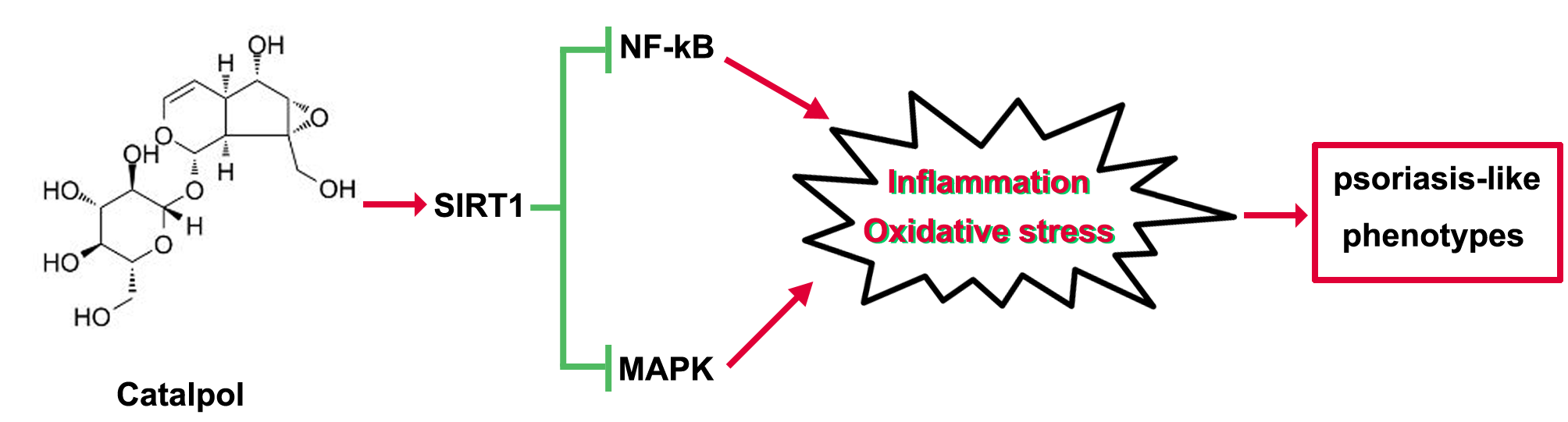

Supplement: Supplemental Material [file KBIE_A_1863015_SM8664.tif]
